# Does Better Health-Related Knowledge Predict Favorable Health Behavior in Adolescents?

**DOI:** 10.3390/ijerph17051680

**Published:** 2020-03-04

**Authors:** Gabriella Nagy-Pénzes, Ferenc Vincze, János Sándor, Éva Bíró

**Affiliations:** 1 Department of Preventive Medicine, Faculty of Public Health, University of Debrecen, 26. Kassai street, 4028 Debrecen, Hungary; penzes.gabriella@sph.unideb.hu (G.N.-P.); vincze.ferenc@sph.unideb.hu (F.V.); sandor.janos@sph.unideb.hu (J.S.); 2 Doctoral School of Health Sciences, University of Debrecen, 26. Kassai street, 4028 Debrecen, Hungary; 3 Division of Health Promotion, Department of Preventive Medicine, Faculty of Public Health, University of Debrecen, 26. Kassai street, 4028 Debrecen, Hungary

**Keywords:** health behavior, health-related knowledge, youth, physical activity, nutrition

## Abstract

The importance of puberty on later health status and behavior is indisputable, which also means that it is worth making intervention efforts during this period of life. However, whether better health-related knowledge is correlated with favorable health behavior in adolescents is an important, still unanswered question. Our objective was to examine this relationship. The participants were ninth-grade secondary school students. Data were collected using anonymous, self-administered questionnaires. The knowledge-related questions were compiled by the authors, while the questions concerning eating habits, physical activity, demographic and socioeconomic data were taken from the Health Behavior in School-Aged Children survey. The relationship between knowledge and behavior was investigated with structural equation modeling adjusted for gender, age, and socioeconomic status. The results demonstrated a good fit to the data, but better knowledge was not related to behavior in our sample. This finding suggests that adolescents’ health behavior is highly influenced by the living context; therefore, appropriate knowledge is necessary but not sufficient to improve adolescents’ behavior. Hence, comprehensive health promotion programs could provide solutions for encouraging healthy behavior.

## 1. Introduction

The importance of puberty on later health status and behavior is indisputable, because this period is associated with an increased risk of many noncommunicable diseases and hazardous behavior. The risks of this period also mean that it is worth making prevention and intervention efforts during puberty, but the interventions have to be tailored to this special target group [1].

One potential tool can be the promotion of health literacy, which is founded on education opportunities throughout the lifespan. However, it is also of utmost importance to help people improve their ability to translate their knowledge into positive health behavior, and health education can be one approach to achieve this [2].

It is an important question whether better health-related knowledge predicts favorable health behavior, therefore well-established evidence is needed to develop effective interventions. The literature reports examples both for a positive association between knowledge and behavior [3,4] and for a knowledge–behavior gap in different populations [5,6], even among health care workers [7,8]. Taking into consideration that adolescents belong to a separate and unique layer of the population, we can assume that the results of studies carried out in other populations will not be fully generalizable to this target group, so we need evidence from studies carried out among adolescents, too. If we search for information related to adolescents in the PubMed database with the search terms “Knowledge” [Mesh] AND “Behavior” [Mesh], we will find only 330 items from the last 40 years [date of search: 22 January 2020]. After screening the titles, it appears that some of the search results are not relevant, and the majority of them discuss sexuality and sexually transmitted diseases. Focusing only on health behavior (i.e., healthy diet [“Knowledge” [Mesh] AND “Diet, Food and Nutrition” [Mesh], filters activated: adolescent: 13–18 years, date of search: 22 January 2020]) and physical activity [“Knowledge” [Mesh] AND “Exercise” [Mesh], filters activated: adolescent: 13–18 years, date of search: 22 January 2020]), fewer than five relevant articles published in the past 30 years can be found.

Therefore, we aimed to investigate the relationship between health-related knowledge and health behavior among secondary school students to fill this gap in knowledge.

## 2. Materials and Methods

### 2.1. Study Population

In this cross-sectional study, we invited ninth-grade students who started their studies in the 2016–2017 and 2017–2018 academic years in a secondary school in Hajdú-Bihar County, Hungary (N = 303). As an inclusion criterion, written parental informed consent was sought, but irrespectively of the given parental consent, the participation of the students occurred on a voluntary basis. The research was performed with the ethical approval of the Medical Research Council Scientific and Research Committee, Hungary (49460-5/2016/EKU).

### 2.2. Data Collection

A total of 303 students were invited to the study. Thirty-nine students refused to participate in the study, and five students were excluded from the second wave because they failed and had already filled out the questionnaire in 2016, so to avoid duplicating data in the database. Therefore, the overall response rate was 85% (N = 259). One case was excluded from the beginning because the majority of answers were missing, and the questionnaire could not be evaluated. The proportion of missing answers (incomplete questionnaires) was approximately one-third of the total number. It took two weeks for all students to complete the questionnaire in the autumn of 2016 and 2017. The study was conducted in classrooms of the participating school, where the first author supervised questionnaire filling. There were no teachers in the classroom to ensure confidentiality. To assess health behavior and health-related knowledge, self-administered, anonymous questionnaires were used. The knowledge-related questions were compiled by the authors, while the questions concerning health behavior (eating habits and physical activity) and demographic and socioeconomic data were taken from the Hungarian version of the Health Behavior in School-Aged Children (HBSC) survey [9].

The following demographic and socioeconomic data were collected: gender, birth year, educational attainment (maximum primary school, vocational certificate, secondary school, university or college), employment status (employed, unemployed) of the mother and father, and family affluence. Family affluence was measured with the ‘Family Affluence Scale’ (FAS III) [10], which can be used to assess material assets in a family. The total score was calculated considering car, computer, and dishwasher ownership; having a bathroom and one’s own bedroom; and the number of family holidays abroad during the last year. The maximum attainable score on the scale is 13 points, and relatively high numbers mean relatively high affluence.

Eating habits were evaluated through five variables, i.e., breakfast and consumption of different types of food (fruits, vegetables, sweets, and sugar-containing soft drinks). In the case of breakfast, the following question was asked: “How often do you usually have breakfast (more than a glass of milk or fruit juice) considering only weekdays?” Response options ranged from ‘never’ to ‘five days’ and were evaluated with a Likert scale. Similarly, the frequency of consumption of fruits, vegetables, sweets, and sugar-containing soft drinks was assessed with a Likert scale from ‘never’ to ‘more than once a day’.

Physical activity was described from two points of view: on one hand, considering the number of days during the last week when the students were so active for at least 1 h in total that their heart rate increased and they were out of breath for a while (moderate-to-vigorous physical activity, MVPA); on the other hand, students were asked how often they usually exercise in their free time (outside to school) to the point of feeling out of breath or sweat (vigorous physical activity, VPA); the answer categories were never, less than once a month, once a month, more than once a month but not every week, once a week, two to three times a week, four to six times a week, and every day.

Health-related knowledge was measured separately for nutrition and physical activity. Regarding nutrition, we asked questions about the importance of breakfast, nutrition pyramid, energy balance, vitamins, and minerals, with a maximum of 31 points. Regarding physical activity, we evaluated students’ knowledge about physical activity recommendations, the advantages of an active lifestyle, the disadvantages of a sedentary lifestyle, the parts of an exercise session, and what happens in the body during exercise, with a maximum of 20 points. A relatively high score meant relatively good health knowledge. The Cronbach’s alpha was 0.59 for the nutrition knowledge test and 0.73 for the physical activity test. The Cronbach’s alpha for the total health-related knowledge test was 0.72.

### 2.3. Data Analysis

The statistical analysis was restricted to participants who provided complete information on all variables included in the analyses.

Descriptive statistics were used to provide an overview of the respondents regarding demographic data, socioeconomic status, and health-related knowledge, using IBM SPSS 25.0. Prior to each analysis, possible outliers were identified, and the distribution and relative variances of the variables were checked.

To investigate the relationship between health-related knowledge and health behavior, we created path models, where the primary outcome variables were moderate-to-vigorous and vigorous physical activity as well as breakfast, fruits, vegetables, sweets, and soft drinks consumption. Health-related knowledge for nutrition and physical activity was investigated as a secondary outcome. The different models were used to describe the relationship between adolescents’ knowledge and health behavior independently from the respondents’ demographic and socioeconomic status.

To test our seven hypothesized models (a separate model for each of the health behavior variables), recursive path analysis models were developed with IBM SPSS AMOS 25.0 to capture the relationship between all variables of interest. The model specifications, including correlations and path justification of the dependent and independent variables, were based on preliminary hypotheses, namely, that during the investigation of the relationship between health behavior and health-related knowledge we cannot ignore the context in which the adolescents are living, which was the reason to involve demographic and socioeconomic variables into the analysis. The latter are widely known health determinants, which can influence health behavior and health-related knowledge, as shown in some previous studies [11,12,13].

After developing the theoretical model, regression paths were created to determine whether health-related knowledge would influence eating habits and physical activity independently from demographic and socioeconomic data, while correlation paths were constructed among independent factors to account for possible intercorrelations and multicollinearity.

Finally, for the examination of modification indices and the revision of model pathways and correlations, seven overidentified recursive path models that retained all of the original hypothesized structures were configured.

Standard guidelines were followed regarding reporting four goodness-of-fit indices; chi-square statistic (χ^2^), comparative fit index (CFI), root-mean-square error of approximation (RMSEA), and *p* of close fit (PCLOSE) were used to evaluate the fitness of the models. A nonsignificant (*p* > 0.05) chi-square statistic indicates that the hypothetical model is well fitted to the data. For the CFI, a value that is greater than 0.95 is desirable. In the case of RMSEA, a value less than 0.05 demonstrates a ‘close fit’, and a value between 0.05 and 0.08 suggests a ‘reasonable fit’ to the data, while PCLOSE is a *p*-value for testing the close-fitting model (i.e., if *p* is greater than 0.05, then the fit of the model is close) [14,15].

Considering the rejection of the assumption of multivariate normality, a bias-corrected (percentile method) bootstrapping procedure (1000 bootstraps) was used to estimate the direct and indirect effects of the explanatory variables.

## 3. Results

### 3.1. Descriptive Statistics

Half of the respondents were female, and the ages ranged from 14 to 16 years among the participants. Nearly one-fifth of the fathers and approximately one-quarter of the mothers had a maximum primary school education. Approximately two-fifth of the fathers and more than one-quarter of the mothers had vocational certificates. Nearly one-fifth of the fathers and more than one-quarter of the mothers finished secondary school, while approximately 5% of the fathers and 8% of the mothers had college degrees. With regard to family affluence, the average FAS score was approximately 5.68. Nearly 90% of the fathers and approximately 80% of the mothers were employed. Descriptive statistics for the whole study population and for the seven outcome variables, separately, are presented in Table 1 and Table 2.

### 3.2. Association between Health Knowledge and Health Behavior Based upon Structural Equation Models

**Figure 1:**
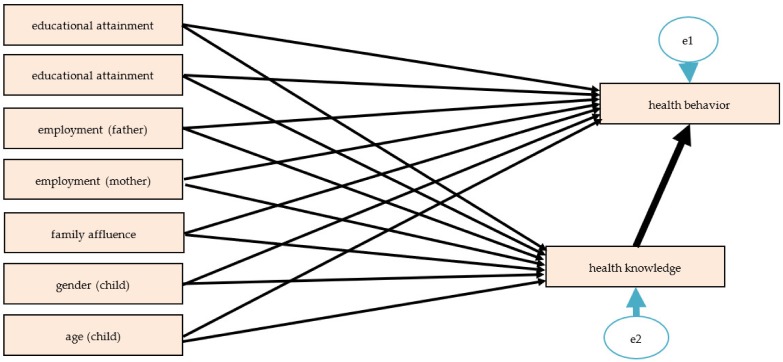
Path model of health-related knowledge and health behavior. Unanalyzed associations: all possible correlations between educational levels, employment, and family affluence and correlations between family affluence and gender (child), educational attainment (father) and gender (child), educational attainment (mother) and gender (child), and educational attainment (mother) and age (child).

Figure 1 presents the structural equation model designed to test the main hypothesis that health-related knowledge is connected to health behavior. As health behavior was measured with seven different variables, a separate model for all of these variables was tested, and the results are presented in Table 3 in detail.

The fit indices indicated that the data fit all models well; all χ^2^ statistics and PCLOSE tests were nonsignificant. The RMSEA (0.049–0.066) and CFI (0.969–0.982) fell below their respective thresholds for acceptable model fit (Table 3).

### 3.3. Models for Nutrition-Related Knowledge and Eating Habits

Based upon the tested models (Models I–V), nutrition knowledge was associated with family affluence, age of the child, educational attainment of the father, and gender, but no relationship was found between knowledge and behavior. The determinants of the behavioral outcomes are described below. Breakfast consumption was less frequent among girls. None of the investigated determinants showed any associations with fruit intake. Among the older students, the frequency of vegetable consumption was lower. There was a negative association between consumption of sweets and educational attainment of the mother, and a higher level of education predicted less frequent eating of sweets. For soft drinks, the same association was found with educational attainment of the mother as in the case of the sweets, and the consumption of soft drinks increased with age (Table 3).

### 3.4. Models for Physical Activity-Related Knowledge and Physical Activity

Based upon the tested models on physical activity (Models VI and VII), only MVPA was associated with knowledge, and the determinants of knowledge and exercise can be seen below. A positive link was found between MVPA and family affluence; girls were less active than boys, and this was the only outcome for which higher physical activity-related knowledge showed a relationship with exercise. Children’s knowledge showed a correlation with educational attainment of the father and was higher among girls.

Similar determinants were detected for VPA, except that the employment status of the mother also influenced the frequency of exercise, while knowledge did not. The level of knowledge was associated with gender; among girls, the achieved scores were higher (Table 3).

## 4. Discussion

Overall, the model fit indices suggested a good fit to the data; however, according to our results, increased knowledge was not associated with the different components of a healthy diet but was influenced by age, gender, father’s education level, and family affluence. Among the older students, the level of nutrition-related knowledge was lower, which may be because these students delayed starting secondary school or had to repeat the ninth-grade, which is usually due to low academic achievement and can lead to a lower nutrition-related knowledge score.

In the case of physical activity, only moderate-to-vigorous physical activity was associated with higher physical-activity-related knowledge. This can be influenced by the fact that for the students, physical education class is obligatory on each school day, and MVPA also includes activities performed in school.

The girls reached higher knowledge scores for both topics, as was expected based on previous studies [16,17,18]. The reason for the inverse association between family affluence and knowledge should be further investigated. The association between knowledge and father’s educational attainment can indicate that the resulting family environment provides increased opportunities for learning experiences [19].

Regarding eating behavior, girls ate breakfast less regularly than boys, which is in line with the results of the Hungarian HBSC survey [9]. The educational attainment of the mother was inversely correlated with the consumption of sweets and soft drinks, which is supported by another study as well [20]. Girls were less active than boys, similarly to the results of the Hungarian HBSC survey [9]. Based on our data, family affluence and parental employment status are associated with a higher level of physical activity, which can be explained by the fact that these families have more opportunities to participate in physical activity by buying the necessary equipment, clothes, season tickets, etc.

Surprisingly, in the literature, a relatively small number of studies can be found regarding the association between knowledge and behavior among adolescents; therefore, the possibility to compare our results with previous results is limited. A positive association has been found between nutrition knowledge and healthy eating in a study among children aged 4–16 years [20], while in a review, the association between food knowledge and dietary intake was not obvious [21]. In a recent intervention study [22], increased knowledge was not translated into sustained behavior change regarding eating habits and physical activity, whereas in another study, a positive association between knowledge and sport activity could not be proven [23]. These results are in line with ours.

### Limitations

This study has limitations that could be further improved in future research. The cross-sectional design is not precise enough to draw clear conclusions in terms of causal effect, but it does provide a foundation for elucidating the relationship. The sample was highly restricted in age, and the participants were from a single high school, so the findings may not be generalizable to other subjects; to obtain a final model, additional research must use community samples. The proportion of the missing answers was approximately equal to one-third of the total number, but due to the low number of cases, the missing answers were not imputed. In addition, information bias may exist in a self-report survey, but this problem cannot be fully avoided, and we can assume that the amount of information bias is similar to that in other surveys.

Despite these limitations, the study has notable strengths. First, the conclusions were drawn based on complex data analyses of a sample of Hungarian adolescents. The sample size was relatively small but was adequate for structural equation model construction, which confirmed that all models tested in the study were acceptably fitted to the data. The applied retrospective data collection followed standardized protocols, and different behaviors were investigated at the same time as suggested in a recent article [24]. Second, the study specifically focused on the complex relationships between adolescent’s health behavior and knowledge. The present findings highlight a number of potentially productive directions for future research, such as finding the most important targets of programs that can improve health behavior and the kind of relationships and interactions that exist between these determinants of health behavior.

Cronbach’s alpha values indicated acceptable internal consistency for the whole knowledge questionnaire as well as, separately, for the physical activity part. The only exception was the section regarding nutrition-related knowledge, but its Cronbach’s alpha was equal to 0.59, indicating a nearly sufficient score; this value could be explained by the mixed type of the questions (e.g., questions about matching the parts of the nutrition pyramid, multiple-choice questions) in the knowledge test. The internal consistency of our questionnaire was similar to that of a published one [25].

## 5. Conclusions

Although appropriate knowledge is necessary, in many cases, it is not sufficient, by itself, to change behavior; adolescents’ living context and school setting are also important. Comprehensive prevention programs can be effective, though mostly for risky behaviors [26,27], when adolescents have the capability to decide about their involvement in certain activities. Regarding health behavior, which in our study encompassed healthy eating and physical activity practice, adolescents’ behavior appeared to be highly influenced by family members as well as habits and opportunities in school, because these factors determine the frequency of eating, the quality of food, and the way adolescents spend their free time. However, it can be hoped that improving students’ knowledge, together with their skills, engaging them in appealing activities, involving their families, and changing their environment, i.e., changing their living context [1], can encourage a healthy behavior.

Schools are cost-effective settings for health education programs, and it is of utmost importance to improve the health knowledge of children and adolescents. However, these kinds of programs can be effective only if they are able to improve the skills of the target group. Therefore, besides informing the students about the features of a healthy lifestyle, they should incorporate practices in order to show low-cost alternatives for healthy eating and being physically active. A program can start with small steps, like promoting water drinking instead of sugary drinks consumption.

Another important thing is the involvement of the parents, as it cannot be assumed that the children will be able to change their behavior without the support of their parents, who are responsible to provide food and monitor children’s free time and are also role models for their children [28].

The school environment is a key factor in health promotion among adolescents as well, as it can support behavior change by making the healthy choice the easy choice by providing healthy food and opportunities and motivation for physical activity. Sometimes, minor changes in the environment are enough to promote health behavior, e.g., decorating the stairs or the schoolyard in a way that will motivate the students to move or improving drinking water sources.

## Figures and Tables

**Table 1:** Demographic and socioeconomic characteristics of the study population (N = 258).

Demographic Indicators		N (%)
Gender of students	male	128 (49.6%)
	female	130 (50.4%)
Educational attainment of father	primary school or less	52 (20.2%)
	vocational school	113 (43.8%)
	secondary school	46 (17.8%)
	university/college	12 (4.7%)
	not known, missing	35 (13.6%)
Educational attainment of mother	primary school or less	65 (25.2%)
	vocational school	71 (27.5%)
	secondary school	72 (27.9%)
	university/college	20 (7.8%)
	not known, missing	30 (11.6%)
Employment status of father	unemployed	8 (3.1%)
	employed	230 (89.1%)
	not known, missing	20 (7.8%)
Employment status of mother	unemployed	40 (15.5%)
	employed	207 (80.2%)
	not known, missing	11 (4.3%)
Age	mean (±SD)	14.9 (0.61)
Family affluence	mean (±SD)	5.68 (2.14)

N: number of cases. SD: standard deviation.

**Table 2:** Descriptive statistics: distribution of determinants for each outcome variable.

		Breakfast Consumption	Fruits Consumption	Vegetables Consumption	Sweets Consumption	Soft Drinks Consumption	Moderate-to-Vigorous Physical Activity	Vigorous Physical Activity
		N (%)	N (%)	N (%)	N (%)	N (%)	N (%)	N (%)
Gender of students	male	89 (46.6%)	88 (46.6%)	88 (46.6%)	88 (46.3%)	89 (46.8%)	83 (46.1%)	79 (48.2%)
	female	102 (53.4%)	101 (53.4%)	101 (53.4%)	102 (53.7%)	101 (53.2%)	97 (53.9%)	85 (51.8%)
Age	14 years old	31 (16.2%)	30 (15.9%)	30 (15.9%)	31 (16.3%)	30 (15.8%)	30 (16.7%)	28 (17.1%)
	15 years old	137 (71.7%)	136 (71.9%)	136 (71.9%)	136 (71.6%)	137 (72.1%)	129 (71.7%)	117 (71.3%)
	16 years old	23 (12.0%)	23 (12.2%)	23 (12.2%)	23 (12.1%)	23 (12.1%)	21 (11.7%)	19 (11.6%)
Educational attainment of father	primary school or less	41 (21.5%)	40 (21.2%)	40 (21.2%)	41 (21.6%)	41 (21.6%)	35 (19.4%)	35 (21.3%)
	vocational school	99 (51.8%)	98 (51.9%)	98 (51.9%)	98 (51.6%)	98 (51.6%)	96 (53.3%)	84 (51.2%)
	secondary school	39 (20.4%)	39 (20.6%)	39 (20.6%)	39 (20.5%)	39 (20.5%)	38 (21.1%)	34 (20.7%)
	university/college	12 (6.3%)	12 (6.4%)	12 (6.4%)	12 (6.3%)	12 (6.3%)	11 (6.1%)	11 (6.7%)
Educational attainment of mother	primary school or less	48 (25.1%)	47 (24.9%)	47 (24.9%)	48 (25.3%)	48 (25.3%)	45 (25.0%)	43 (26.2%)
	vocational school	60 (31.4%)	59 (31.2%)	59 (31.2%)	59 (31.1%)	60 (31.6%)	57 (31.7%)	50 (30.5%)
	secondary school	65 (34.0%)	65 (34.4%)	65 (34.4%)	65 (34.2%)	64 (33.7%)	60 (33.3%)	53 (32.3%)
	university/college	18 (9.4%)	18 (9.5%)	18 (9.5%)	18 (9.5%)	18 (9.5%)	18 (10.0%)	18 (10.9%)
Employment status of father	unemployed	6 (3.1%)	6 (3.8%)	6 (3.2%)	6 (3.2%)	6 (3.2%)	6 (3.3%)	5 (3.1%)
	employed	185 (96.9%)	183 (96.8%)	183 (96.8%)	184 (96.8%)	184 (96.8%)	174 (96.7%)	159 (96.9%)
Employment status of mother	unemployed	30 (15.7%)	30 (15.9%)	30 (15.9%)	30 (15.8%)	29 (15.3%)	26 (14.4%)	25 (15.2%)
	employed	161 (84.3%)	159 (84.1%)	159 (84.1%)	160 (84.2%)	161 (84.7%)	154 (85.6%)	139 (84.8%)
Total		191 (100.0%)	189 (100.0%)	189 (100.0%)	190 (100.0%)	190 (100.0%)	180 (100.0%)	164 (100.0%)
Family affluence	mean (±SD)	5.80 (2.18)	5.81 (2.18)	5.81 (2.18)	5.79 (2.19)	5.79 (2.19)	5.79 (2.16)	5.73 (2.13)
Health knowledge (nutrition or physical activity)	mean (±SD)	15.00 (4.66)	15.74 (4.68)	15.74 (4.68)	15.78 (4.64)	15.77 (4.66)	15.49 (3.26)	15.49 (3.31)
Proportion of missing answers		25.9%	26.7%	26.7%	26.4%	26.4%	30.2%	36.4%

N: number of cases. SD: standard deviation.

**Table 3:** Structural equation models^ of health-related knowledge and health behavior.

	MODEL I.			MODEL II.			MODEL III.		
	Health Knowledge (Nutrition)	Breakfast Consumption		Health Knowledge (Nutrition)	Fruits Consumption		Health Knowledge (Nutrition)		Vegetables Consumption
Employment of mother (ref.: employed)	−0.03 [*p* = 0.523]	0.06 [*p* = 0.493]		−0.10 [*p* = 0.124]	0.01 [*p* = 0.864]		−0.05 [*p* = 0.505]		−0.03 [*p* = 0.478]
Employment of father (ref.: employed)	−0.05 [*p* = 0.083]	−0.02 [*p* = 0.801]		−0.04 [*p* = 0.605]	0.01 [*p* = 0.852]		−0.10 [*p* = 0.169]		−0.02 [*p* = 0.722]
Family affluence	**−0.08 [*p* = 0.031]**	0.03 [*p* = 0.720]		**−0.17 [*p* = 0.030]**	0.08 [*p* = 0.159]		**−0.17 [*p* = 0.025]**		0.12 [*p* = 0.082]
Educational attainment of mother (ref.: primary or less)	−0.10 [*p* = 0.085]	−0.12 [*p* = 0.262]		−0.15 [*p* = 0.162]	0.02 [*p* = 0.797]		−0.16 [p = 0.081]		−0.04 [*p* = 0.775]
Educational attainment of father (ref.: primary or less)	**0.21 [*p* = 0.002]**	−0.06 [*p* = 0.583]		**0.38 [*p* = 0.012]**	−0.01 [*p* = 0.892]		**0.38 [*p* = 0.008]**		−0.05 [*p* = 0.495]
Age (child)	**−0.21 [*p* = 0.009]**	−0.09 [*p* = 0.460]		**−0.42 [*p* = 0.004]**	−0.01 [*p* = 0.955]		**−0.42 [*p* = 0.034]**		**−0.24 [*p* = 0.009]**
Gender (child, ref.: boy)	**0.24 [*p* = 0.008]**	**−0.41 [*p* = 0.027]**		**0.48 [*p* = 0.003]**	−0.10 [*p* = 0.329]		**0.49 [*p* = 0.030]**		0.11 [*p* = 0.334]
Health knowledge (nutrition)		−0.09 [*p* = 0.529]		.	0.05 [*p* = 0.179]		.		0.06 [*p* = 0.065]
Fit statistics of the model	χ^2^(df) = 10.390 (7); χ^2^(*p*−value) = 0.168; CFI = 0.979; RMSEA = 0.050; PCLOSE = 0.431			χ^2^(df) = 10.319 (7); χ^2^(*p*−value) = 0.171; CFI = 0.979; RMSEA = 0.050; PCLOSE = 0.434			χ^2^(df) = 10.340 (7); χ^2^(*p*−value) = 0.170; CFI = 0.980; RMSEA = 0.050; PCLOSE = 0.432		
	**MODEL IV.**					**MODEL V.**			
	**Health Knowledge (Nutrition)**		**Sweets Consumption**			**Health Knowledge (Nutrition)**		**Soft Drinks Consumption**	
Employment of mother (ref.: employed)	−0.05 [*p* = 0.534]		0.07 [*p* = 0.358]			−0.02 [*p* = 0.771]		0.01 [*p* = 0.979]	
Employment of father (ref.: employed)	−0.11 [*p* = 0.103]		0.10 [*p* = 0.333]			−0.11 [*p* = 0.082]		0.10 [*p* = 0.377]	
Family affluence	**−0.17 [*p* = 0.037]**		−0.04 [*p* = 0.460]			−**0.16 [*p* = 0.043]**		−0.07 [*p* = 0.368]	
Educational attainment of mother (ref.: primary or less)	−0.16 [*p* = 0.179]		**−0.17 [*p* = 0.019]**			−0.14 [*p* = 0.226]		−**0.18 [*p* = 0.029]**	
Educational attainment of father (ref.: primary or less)	**0.38 [*p* = 0.004]**		0.04 [*p* = 0.602]			**0.37 [*p* = 0.012]**		−0.13 [*p* = 0.168]	
Age (child)	**−0.42 [*p* = 0.013]**		0.07 [*p* = 0.474]			−**0.45 [*p* = 0.003]**		**0.27 [*p* = 0.038]**	
Gender (child, ref.: boy)	**0.47 [*p* = 0.002]**		0.08 [*p* = 0.495]			**0.51 [*p* = 0.004]**		0.06 [*p* = 0.643]	
Health knowledge (nutrition)	.		−0.04 [*p* = 0.393]			.		−0.08 [*p* = 0.165]	
Fit statistics of the model	χ^2^(df) = 10.146 (7); χ^2^(*p*−value) = 0.180; CFI = 0.982; RMSEA = 0.049; PCLOSE = 0.448					χ^2^(df) = 10.484 (7); χ^2^(*p*−value) = 0.163; CFI = 0.982; RMSEA = 0.051; PCLOSE = 0.423			
	**MODEL VI.**					**MODEL VII.**			
	**Health Knowledge (Physical Activity)**		**Moderate−to−Vigorous Physical Activity**			**Health Knowledge (Physical Activity)**		**Vigorous Physical Activity**	
Employment of mother (ref.: employed)	−0.03 [*p* = 0.670]		−0.05 [*p* = 0.500]			−0.08 [*p* = 0.226]		**−0.21 [*p* = 0.036]**	
Employment of father (ref.: employed)	−0.02 [*p* = 0.725]		−0.14 [*p* = 0.143]			−0.06 [*p* = 0.410]		−0.21 [*p* = 0.057]	
Family affluence	0.05 [*p* = 0.331]		**0.18 [*p* = 0.044]**			0.09 [*p* = 0.122]		**0.16 [*p* = 0.023]**	
Educational attainment of mother (ref.: primary or less)	0.01 [*p* = 0.926]		−0.04 [*p* = 0.692]			0.00 [*p* = 0.984]		−0.05 [*p* = 0.602]	
Educational attainment of father (ref.: primary or less)	**0.24 [*p* = 0.028]**		0.06 [*p* = 0.604]			**0.25 [*p* = 0.013]**		0.07 [*p* = 0.476]	
Age (child)	−0.08 [*p* = 0.470]		−0.22 [*p* = 0.083]			−0.09 [*p* = 0.431]		0.02 [*p* = 0.889]	
Gender (child, ref.: boy)	**0.28 [*p* = 0.030]**		**−0.54 [*p* = 0.002]**			**0.27 [*p* = 0.047]**		**−0.50 [*p* = 0.001]**	
Health knowledge (physical activity)			**0.20 [*p* = 0.025]**					0.13 [*p* = 0.111]	
Fit statistics of the model	χ^2^(df) = 12.531 (7); χ^2^(*p*−value) = 0.084; CFI = 0.969; RMSEA = 0.066; PCLOSE = 0.277					χ^2^(df) = 11.972 (7); χ^2^(*p*−value) = 0.101; CFI = 0.972; RMSEA = 0.066; PCLOSE = 0.290			

Significant associations are marked in bold. ^ regression weights [*p*-value]. ref.: reference category. χ^2^(df) = model chi-squared statistic (degrees of freedom). χ^2^(*p*-value) = *p*-value of the exact-fit hypothesis. CFI = comparative fit index. RMSEA = root-mean-square error of approximation. PCLOSE = *p*-value for the close-fit hypothesis.
